# Supplementary material for: Engineered cell‐to‐cell signalling within growing bacterial cellulose pellicles
Source: Microb Biotechnol. 2018 Nov 21;12(4):611–9. doi: 10.1111/1751-7915.13340 (PMC6559020; doi:10.1111/1751-7915.13340)
Supplement: Supplementary file 1 — Fig. S1. Dose response of E. coli Receiver cells induced with increasing concentrations of synthetic AHL.Fig. S2. Growth curve showing that buffered HS media (grey), and spent media sourced from K. rhaeticus strains with the Sender (red) or empty (black) plasmids do not alter E. coli growth.Fig. S3. Flow cytometry data of the induced and uninduced K. rhaeticus Receiver strains. [file MBT2-12-611-s001.docx]

**Supplementary Materials**

**Supplementary Figure 1.** Dose response of *E. coli* Receiver cells induced with increasing concentrations of synthetic AHL. Points represent the mean mRFP production rate of 3 replicates for each AHL concentration 1 hour after induction. Error bars represent the standard deviation of the 3 replicates.

**Supplementary Figure 2.** Growth curve showing that buffered HS media (grey), and spent media sourced from *K. rhaeticus* strains with the Sender (red) or empty (black) plasmids do not alter *E. coli* growth. Points represent mean OD_600_ values of 3 replicates with the exception of buffered HS media, which represents 2 replicates. Error bars represent standard deviation of the replicates at each time point.


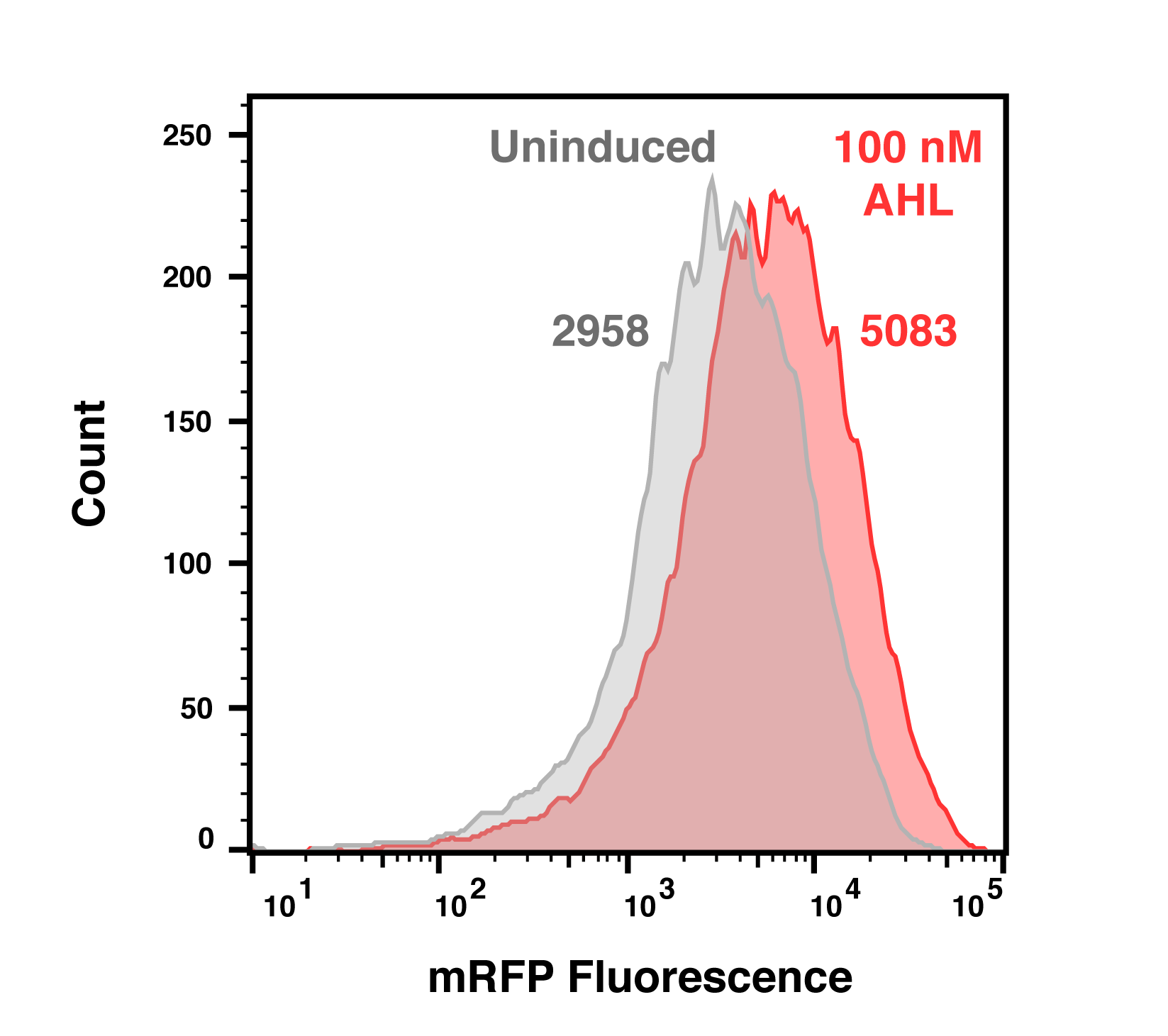


**Supplementary Figure 3.** Flow cytometry data of the induced and uninduced *K. rhaeticus* Receiver strains. Fluorescence was measured 4 hours post induction with 100 nM synthetic AHL - excitation 561 nm and emission 620(15) nm were used to measure mRFP. The geometric mean fluorescence value is displayed in grey for the uninduced cells and in red for the cells induced with 100 nM AHL. The uninduced gate contained 15725 cells whilst the 100 nM AHL gate contained 16430 cells. The experiment was conducted in triplicate with results being observed across repeats.

**Supplementary Methods**: **Flow cytometry of induced and uninduced *K. rhaeticus* Receiver**

To record single cell data on the induction dynamics of *K. rhaeticus* Receiver, flow cytometry was conducted. *K. rhaeticus* Receiver cells were grown shaking in HS media with 2% cellulase to an OD_600_ of 0.3. After this initial growth, the cells were passaged in a 1:50 dilution to 10 mL HS media with 2% cellulase and again grown shaking at 30°C. Once *K. rhaeticus* Receiver cells reached an OD_600_ of 0.3, half of the cultures were induced with 100 nM AHL and grown with shaking at 30°C. After 4 hours 100 μL of cell culture was taken from the uninduced and induced cultures and diluted into 900 μL of PBS buffer pH 7.4. Single cell fluorescence data was recorded with the Attune NxT flow cytometer (Thermo Scientific) with the following settings for measuring mRFP: FSC 440 V, SSC 440 V, YL2 400 V. Single cell fluorescence data was collected from ~16000 cells for each experiment and analysed using FlowJo software.
